# KNEE SYNERGISM DURING GAIT REMAIN ALTERED ONE YEAR AFTER ACL RECONSTRUCTION

**DOI:** 10.1590/1413-785220162403153479

**Published:** 2016

**Authors:** GUSTAVO LEPORACE, LEONARDO METSAVAHT, GLAUBER RIBEIRO PEREIRA, LISZT PALMEIRA DE OLIVEIRA, BERNARDO CRESPO, LUIZ ALBERTO BATISTA

**Affiliations:** 1. Universidade Federal do Rio de Janeiro, Rio de Janeiro, RJ, Brazil.; 2. Instituto Brasil de Tecnologias da Saúde(IBTS), Rio de Janeiro, RJ, Brazil.; 3. Universidade do Estado do Rio de Janeiro, Rio de Janeiro, RJ, Brazil.; 4. Instituto Nacional de Traumatologia e Ortopedia (INTO), Rio de Janeiro, RJ, Brazil.

**Keywords:** Anterior cruciate ligament reconstruction, Electromyography, Gait.

## Abstract

**Objective:**

To compare the activation of the vastus lateralis (VL) and biceps femoris (BF) muscles during gait, as well VL/BF muscular co-contraction (MCC) between healthy (CG) and anterior cruciate ligament reconstructed (ACL-R) subjects.

**Methods:**

Nineteen subjects, ten controls and nine ACL-R patients had a VL and BF electromyogram (EMG) captured to calculate the MCC ratio. A Principal Component (PC) Analysis was applied to reduce the dimensionality effect of each of the MCC, VL and BF curves for both healthy and ACL reconstructed groups. The PC scores were used to calculate the standard distance (SD). SD values were employed in order to compare each dependent variable (MCC, VL and BF) between the two groups using unpaired t-test.

**Results:**

ACL-R group presented a lower VL activation at the beginning and at the end of the gait cycle, as compared to the control group. However, no difference was found for BF or VL/BF MCC.

**Conclusion:**

The gait analysis of ACL reconstructed patients demonstrated a persistent deficit in VL activation when compared to the control group, even one year after surgery. ***Level of Evidence III. Case Control Study***

## INTRODUCTION

The tear of the anterior cruciate ligament (ACL) is one of the most common injuries to knee joint, occurring mainly in sports and recreational activities, with over 100.000 reconstructions per year performed in the United States.^1^ The aim of this surgery is to restore the stability of the knee and allow return to pre-injury activity levels. However, early joint degeneration has been reported even after an ACL reconstruction.^2^ The presence of high joint compressive forces and changes in gait biomechanics has been suggested as potential mechanisms for increasing the risk of early onset of osteoarthritis development.^3^ ^-^ ^4^

Besides the biomechanical changes after ACL reconstruction, neuromuscular adaptations for daily activities may be associated with the early degenerative process. Changes on the synergism between quadriceps and hamstrings^5^ seem to have an important role on these changes. Zebis et al.^6^ suggested that reduced activity of hamstrings in relation to quadriceps can predispose to ACL rupture. Furthermore, an excessive activation of quadriceps with a low activation of hamstrings muscles leads to an excessive anterior shear load on the knee, increasing ACL strain, indicating that hamstrings have an important role as a neuromuscular ACL agonist.

Several authors have analyzed the electromyogram (EMG) of lower limb muscles of patients who have had ligament reconstruction among different tasks and intensities.^4^ ^,^ ^7^ However, few studies have analyzed the muscular co-contraction (MCC) of these muscles during gait. Lustosa et al.^7^ reported that the MCC of quadriceps and hamstrings during single limb stance is lower in surgically-repaired limb than non-repaired limb. In addition, deficits in the functional outcome were found in subjects after one year of ACL reconstruction surgery.^8^ Thereby, this may be one reason to explain why some subjects are unable to return to their pre-injury activity level after undergoing ACL surgery and also have been at higher risk of a new injury to the previously injured or the contralateral ACL.^9^

The muscular co-contraction is a phenomenon characterized by the simultaneous contraction of any two or more muscles surrounding a joint. This phenomenon is considered essential for the regulation of muscle stiffness and the maintenance of dynamic joint stability.^10^ Nonetheless, the mechanism utilized by ACL-reconstructed subjects to stabilize knee joint during gait is still not well understood.

Therefore, this study aimed at comparing the myoelectric activity of the vastus lateralis and biceps femoris and the co-contraction between these two muscles among healthy and ACL reconstructed subjects. The hypothesis was that vastus lateralis, biceps femoris and the co-contraction between these muscles would still be different at the time of the comparisons.

## MATERIALS AND METHODS

Nineteen subjects, 10 in the control group (CG) and nine in the anterior cruciate ligament reconstruction group (ACL-R), with similar anthropometric characteristics participated in this study. (Table 1) The ACL-R group had undergone single-bundle ACL reconstruction using hamstring tendon autografts. All of them presented a complete ACL tear evidenced by magnetic resonance imaging and a positive pivot-shift test under anesthesia and confirmed by direct visualization at the arthroscopic procedure. All ACL-R subjects had unilateral ligament tear, with no prior ligament injury or previous knee surgery. All surgeries were performed by the same surgeon. The mean time between surgery and biomechanical gait analysis was 11.2 ± 2.4 months (ranging between 8 and 15 months). All patients underwent similar rehabilitation programs and presented a range of motion within normal range at the time of the test.

**Table 1:** Anthropometric data of the sample.

	CG	ACL-R
Age (years old)	29.4 ± 3.1	33.1 ± 11.1
Height (cm)	178.4 ± 4.1	182.3 ± 2.9
Body Mass (kg)	79.1 ± 7.3	82.1 ± 7.4

CG: Control group; ACL-R: Anterior Cruciate Ligament Reconstructed group. mean ± standard deviation.

The inclusion criteria for the CG were subjects between 20 and 40 years of age, scoring over 90% of the maximum in the subjective evaluation questionnaires *International Knee Documentation Committee* (IKDC) *Subjective Knee Form* and *Lower Extremity Functional Scale*.^11^ ^,^ ^12^ Subjects with a history of neurological and orthopedic injuries and lower limb pain were excluded from the CG. All participants signed an informed consent form allowing participation in the study. This study was approved by the Ethics Research Council of *Universidade Federal do Rio de Janeiro* under number 053/2009.

Subjects were instructed to walk at a self-selected speed on an 8 meter long walkway. Each subject performed six laps. The first three laps were not collected to allow familiarization with the task. The last three laps were evaluated to determine the muscle electrical activity of right lower limb in the CG group and the injured limb in the ACL-R group during three gait cycles.

The myoelectric activity analysis was performed using surface electromyography techniques. The signals were captured using Acknowledge software version 3.5 (TEL 100D, BIOPAC System, Santa Barbara, USA) with a bipolar differential amplifier (input impedance: 2 MΩ, Common Mode Rejection Ratio > 110 db, gain: 1000), and converted from analog to digital (1.8 kHz, 12 bit, MP100WSW, BIOPAC Systems).

Ag/AgCl electrodes (Kobme, Protect Bio, Korea) were positioned on the vastus lateralis (VL) and biceps femoris (BF). The VL electrodes were placed 5 cm distant of the lateral border of the patella at an oblique angle. The BF ones were positioned in the lateral thigh, at two-thirds the distance between the trochanter and lateral condyle of the femur. The electrodes were placed parallel to the muscle fibers, with an inter electrode distance of 2 cm. The reference electrode was placed on the seventh cervical vertebra spinous process.

Before electrodes placement, the skin was prepared by shaving the area and cleansing it with alcohol to reduce surface impedance. Electrode cables were fixed to the skin using adhesive tape (3M Ltda, Brazil) in order to prevent movement artifacts in the signals.

To determine the time interval of each gait cycle, two footswitches were positioned (FootPress, LaBiCoM), one in the heel area and another under the first metatarsal head of the analyzed limb. When each region of the foot was in contact with the ground, the circuit generated an electrical signal captured by a BIOPAC (UMI 100B, BIOPAC Systems), which was then synchronized with the EMG data to determine the exact moment of ground contact.

The raw EMG signals from three cycles of each muscle were filtered using a 2^nd^ order Butterworth filter (20 - 400 Hz) applied in the direct and reverse directions to avoid phase distortions. The resultant signal was rectified and filtered again by a low-pass 2^nd^ order recursive Butterworth filter with cut-off frequency of 12 Hz. The signals were normalized by the arithmetic mean of the three highest peaks found in all three cycles and processed in a manner similar to that described above.

The muscular co-contraction (MCC) temporal magnitude was determined throughout the value of the common area between the curves of normalized EMG of the VL and BF regarding each gait cycle. The area of intersection between these curves represents the intensity of simultaneous muscle activation.^7^ ^,^ ^13^ After obtaining the VL and BF envelope curves and the MCC curve, each signal was interpolated to 51 points in order to represent from 0% to 100% of the gait cycle. The signals were processed by means of the software Matlab 7.04 (The Mathworks, USA).

### Statistical Analysis

The EMG signals (VL, BF and MCC) in the control group and ACL-R were concatenated into three matrices E [19 x 51], where the rows corresponded to the subjects in each group and the columns to the EMG signal of an interpolated gait cycle. A principal components analysis (PCA) was applied to each of these matrices, independently, to reduce data dimensionality. For this purpose, the mean of each E column was removed, the covariance matrix S [51 x 51] was calculated and, finally, the eigenvectors and eigenvalues were estimated based on solution of the following linear system:



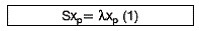



where λ is the eigenvalues of S, arranged in descending order, and x_p_ are the correspondent eigenvectors. The linear system was solved based on a singular value decomposition algorithm, as described below, 



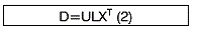



where E is the matrix with the original dataset, the columns of U are called the left singular vectors, the rows of X^T^ are the right singular vectors and the L is a diagonal vectors whose nonzero entries are the singular values. X, L and U contain, respectively, the eigenvectors, the square root of the eigenvalues of and the principal components (PC) scores.

The number of PC retained in the analysis from each EMG data were those that the cumulative sum accounted approximately 80% of the original data variance.^3^ The PC scores retained were used to calculate the Standard Distance (SD). The SD is the square root of the Mahalanobis distance, and corresponds to the distance between each subject of the ACL-R group in relation to the centroid of the PC scores of the CG, normalized by its standard deviation:



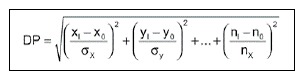



where s_x_,s_x_,s_n_ are the standard deviations from the first, second and nth PC, respectively; x_i_, y_i_, n_i_ are the scores of the first, second and nth PC, respectively, while i is the number of subjects; and x_o_, y_o_, n_o_ are the average of the scores of the first, second and nth PC, respectively.

The unpaired t test was employed in order to compare the SD from VL and BF signal and MCC between the two groups. This test was selected since the adherence tests (Shapiro-Wilk and Kolmogorov-Smirnov) ratified the Gaussian distribution of the data. The significance level was 0.05. The effect size was calculated for all variables and values greater than 0.8 were considered high, and those below 0.5 were considered low. The software Matlab 7.04 (The Mathworks, USA) was used to run the PCA and the t tests were performed with the GraphPad Prism, Version 5.0 (GraphPad Software, San Diego, California, USA).

The most important PCs retained in the analysis, for the comparisons in which statistical differences were found, were analyzed in temporal correspondence to the original signals of both groups to identify the location where the variance between them could be explained.^3^ ^,^ ^14^ Locations where PCs deviate from zero indicate increased differences between groups.

## RESULTS

**Figure 1:**
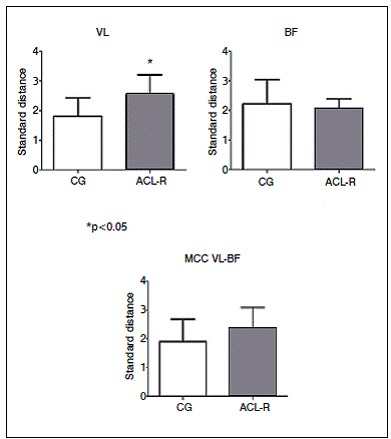
Standard Distance of vastus lateralis (VL), biceps femoris (BF) and muscular co-contraction (MCC VL-BF) of both groups. CG: Control Group; ACL-R: Anterior Cruciate Ligament Reconstructed group.

Five PC were retained in each of the three PCA performed corresponding to 82.7%, 81.4% and 84.7% of the VL, BF and MCC signals variance, respectively. The unpaired t test indicated that the ACL-R group showed higher DP values than CG in the VL activity (p = 0.022; CG: 1.80 ± 0.63; ACL-R: 2.54 ± 0.66, effect size: 1.15). For the BF (p = 0.611; CG: 2.22 ± 0.81; ACL-R: 2.07 ± 0.31, effect size: 0.25) and the MCC (p = 0.236; CG: 1.90 ± 0.77; ACL-R: 2.33 ± 0.76, effect size: 0.56), there were no significant differences. (Figure 1)

**Figure 2:**
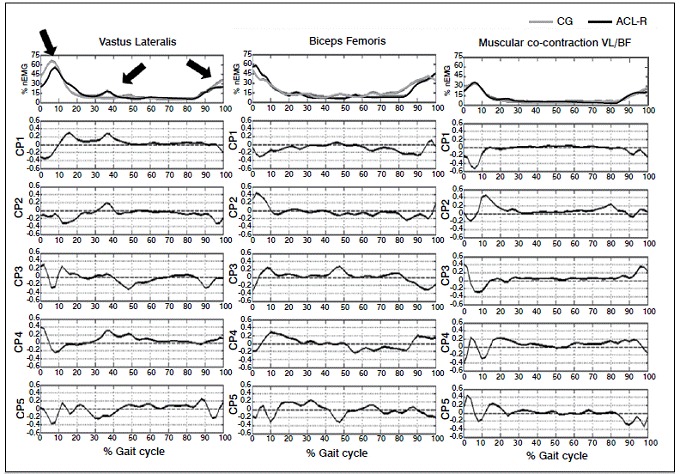
Myoelectric activity of vastus lateralis (left), biceps femoris (center) and BF/VL co-contraction (right). The first row represents the average of the control group (gray line) and ACL-R group (black line). The second to fifth lines represent the principal components retained in the analysis. The CP1 to CP5 arrows in the upper graph indicate the locations where the retained PCs identified the most important differences in the gait cycle for the vastus lateralis. nEMG: % normalized EMG; PC: Principal Component.

The analysis of the retained PC for VL (Figure 2) indicated that one of the main differences is located in the initial 10% of the gait cycle, corresponding to the load response phase, in which CG showed higher signal amplitude than the ACL-R. Another difference identified by the PC of VL was the increased muscle activation around 40% of the gait cycle for ACL-R, whereas this raise was only observed around 50% of the cycle in CG. Finally, some PC has also identified that the difference in load response starts at the end of the balance (around 90% of the gait cycle), and that the CG had higher activation levels than ACL-R, suggesting an increased recruitment at the beginning of the cycle. Figure 2 also shows the biceps femoris activation and VL/BF muscular co-contraction.

## DISCUSSION

The purpose of this study was to compare the myoelectric activity of the vastus lateralis, biceps femoris and the co-contraction of these two muscles between healthy and anterior cruciate ligament reconstructed subjects. The hypothesis of different activation of these muscles was partially confirmed by the results of this study.

Joint stability is maintained mainly by passive and active structures surrounding the joints, regulating the dynamic stiffness. In the past few decades researches have pointed out that most of these structures have afferent receptors and that act improving the dynamic stability of the joint, mainly by two mechanisms, denominated ligament muscular reflex (feedback) and anticipatory preparation adjustments of muscle stiffness via gamma-muscle spindle system (feed forward).

The ligament muscular reflex is related to the increase of excitability of alpha motoneurons in response to an increased strain on a ligament. For instance, when the ACL is subjected to forces that excessively displace the tibia anteriorly, receptors in the ACL trigger hamstrings reflex protective contractions pulling the tibia posteriorly. Feed forward mechanisms involve the activation of muscles before an event even happens, preparing the joint to deal with external perturbations by increasing stiffness. This mechanism is modulated by gamma muscle-spindle system initiated by ligament and articular receptors.^15^

Baratta et al.^16^ proposed that simultaneous contraction of antagonist muscle groups around a joint would not only result in greater joint stiffness but would also increase and regulate the contact force between joint surfaces. Decreased and increased joint stiffness should be avoided, since the former can lead to chondral shear stress through joint instability, while the latter can lead to excessive chondral compression, and both situations are related to joint degeneration.^4^ Therefore, the study of EMG signals of muscles during daily living activities is of extreme relevance since it provides an alternative to verify the different strategies that injured subjects assume to compensate for ligament injuries.

With regard to the knee, ACL injuries may decrease or completely abolish the afferent information from the ligament receptors. This is believed to be strongly related to an increased rate of re-injury and early joint degeneration, usually seen in these patients.^9^ Several studies have showed that even one year after ACL reconstruction, patients still have altered knee biomechanics during gait and landing tasks.^3^ ^,^ ^13^ ^,^ ^16^ In the present study, it was found that the myoelectric activity is disturbed after ligament reconstruction and rehabilitation process.

Excessive activation of vastus lateralis is associated to an increased displacement of the tibia in relation to the femur and generating an increase in the anterior shear forces.^17^ To counteract this action, hamstrings muscles are activated to control knee extension torque and maintain joint homeostasis. In the present study, no myoelectric differences were verified in the biceps femoris of the ACL-R group but a clear decrease on vastus lateralis activity was found in the terminal swing phase and beginning of loading response. (Figure 1) The co-contraction between these muscles was preserved. Therefore, it seems that the alternative way found by the central nervous system was to decrease vastus lateralis activity before initial ground contact, supposedly due to the absence of ligament mechanoreceptors. This altered coordination pattern seemed to be a strategy to keep muscle stiffness within the limits of non-injured subjects, as showed in the results of the present study. (Figure 2)

Several authors have already evidenced the decrease in the vastus lateralis and increase in the biceps femoris activity in anterior cruciate ligament deficient subjects.^18^ Some studies have proposed that after ligament reconstruction it takes at least eight months to find a normal EMG trace.^19^ However, these studies used parametric variables to analyze EMG between injured and non-injured groups. In the present study, data from the whole gait cycle was compared, by application of principal component analysis, allowing a more sensitive detection of changes in myoelectric activity.^14^

A few studies have compared the co-contraction during gait in ACL-R subjects, while a great number of studies have compared healthy and ACL deficient subjects.^13^ ^,^ ^20^ Our findings in ACL-R subjects support the one from Lustosa et al.^7^ Although study designs were different, the co-contraction level between quadriceps and hamstrings observed in the involved limbs of the ACL-R group was similar to that observed in individuals without ligament injury.^1^ ^,^ ^13^

The differences observed during midstance phase (Figure 1) for the vastus lateralis should be related to impairment in the proprioception, altering the timing of activation of the vastus lateralis, which is commonly seen in the beginning of the pre-swing phase. Further studies should be carried out to measure the consequences of these changes on the knee stability.

One limitation of this study was the sample size, for that reason we minimized possible bias by analyzing the sample distribution and using the effect size to complement the unpaired t-test. Future studies are necessary to monitor the dynamic joint stability by means of the variables used in this study, either during the rehabilitation process as well as for long term after surgery.

## CONCLUSION

Even one year after anterior cruciate ligament reconstruction some differences in myoelectric activity of the thigh muscles were still present and possibly related to protective strategies to avoid excessive tibial shear forces originated from vastus lateralis activity.
